# Case of Ovariotomy—Successful

**Published:** 1862-09

**Authors:** A. Fisher

**Affiliations:** Chicago


### ORIGINAL COMMUNICATIONS.

### CASE OF OVARIOTOMY—SUCCESSFUL.

Reported by A. FISHER, M. D., Chicago.

Mrs. Thinius, a German lady, aged 47, of a bilious and nervous temperament, has had seven children, the youngest 14 years old; menstruation ceased 9 or 10 years since. First discovered a small tumor in the right side about a year ago. She said, however, that it soon disappeared, and about the same time she detected one in the left iliac region, where it has remained ever since, gradually increasing in size.

At that time, July 8th, 1862, she was much larger than women ordinarily are just before parturition. An indurated tumor could be distinctly felt in the left iliac region, circular in form and about six inches in diameter. It was movable in every direction and could be separated from the uterus two or three inches. Although the parietes of the abdomen were very much distended, hard and unyielding, fluctuation was perceptible in every part of the abdomen, with the exception of the space occupied by the tumor. She has never experienced any pain or inconvenience from it, except from pressure for the past two or three weeks.

The case was diagnosed, by Dr. Myers and myself, ovarian tumor with ascites; Prof. Allen afterwards saw the case with us, and confirmed our diagnosis. We told the patient that she might get temporary relief by tapping; but the only hope of a permanent cure was extirpation of the tumor, and that would be a very severe and dangerous operation. After telling her the facts and even magnifying the danger, she concluded to have the operation performed.

Accordingly, on the 10th of July, after evacuating the patient's bowels freely, she was placed upon a table properly prepared, and soon brought under the influence of chloroform by Professor Allen, who kept her fully anæsthetized, carefully watching her during the operation, which was performed in the presence of Drs. Myers, Macalister and Rogers, who assisted me. The operation was commenced by making an incision in the linea alba below the umbilicus two or three inches in length into the peritoneal cavity. Two or three gallons of clear serum was evacuated from the peritoneal cavity, and three or four quarts from the sack attached to the tumor. Finding no adhesion, the opening was enlarged to about six inches in length. I then introduced my hand and drew out the tumor with the sack, and passed a needle around with a double ligature through the pedicle, as near the tumor as possible, leaving enough exterior to it when separated, to prevent the ligatures from slipping off. The ligatures were strong and tied very tight. The tumor was then cut off and the pedicle kept exterior to the wound by an assistant. After examining the right ovary, and finding it healthy, I carefully sponged out what serum remained, and brought the edges of the wound together, and retained them in position by deep sutures, being careful not to include the peritoneum. The pedicle was then confined to the lower edge of the wound, by a stitch through it and the abdominal integuments, leaving the ligatures exterior to the wound. Adhesive plasters were then applied, and the wound dressed with lint compress and bandage, and the patient carefully placed in bed.

On examining the parts removed, we found the ovary very much indurated, and about the size of a small hen's egg, and attached to a tumor circular in form, and about six inches in diameter, two inches thick in the center, rounded on the external and flat on the internal surface, so as to form an edge at the circumference, with a thick membrane proceeding from the edge of the tumor, forming a sack which contained three or four quarts of serum. The tumor with the sack very much resembled in form the placenta with its membranes attached. Both surfaces of the tumor and sack were smooth and appeared like the continuation of the peritoneal membrane thickened. The structure of the tumor was fibrous and very much indurated.

As soon as the effect of the chloroform passed off, after the operation, a full dose of opium was given to confine the bowels and allay irritation. Her pulse was then 65 and rather weak. I remained with her an hour; during that time her pulse came up to 75. As she was very comfortable, I left her. Dr. Myers saw her with me at 4 P. M.; reaction was fully established; pulse 100, soft and full; rather restless, but complains of no pain; gave morphine half grain, and ordered ice water in teaspoonful doses to wet her mouth. 10 P. M.—Pulse soft, full and regular. Slight efforts to vomit. Drew off 6 or 8 oz. of urine with the cathether. Prescribed a full dose of morphine, and continued ice water.

11th, 9 A. M.—Pulse 95; skin nearly natural; tongue slightly coated; voided 4 or 5 oz. urine. Complains very little of pain or tenderness of the bowels. ℞—Morphine gr. ss, if she has pain or is restless; diet, rice and barley water in small quantities. 4 P. M.—Pulse 90, soft and full; drew 8 or 10 oz. urine. ℞—Morphine gr. ss, if necessary to keep her quiet.

12th, 9 A. M.—Rested well last night; pulse 90, soft, full and regular; bowels neither tender nor tympanitic; tongue moist; no thirst; every way comfortable. Evacuated the bladder and continue morphine if necessary. 10 P. M.—Pulse 80. She has some appetite, has taken two or three small crackers in coffee. Evacuated the bladder; urine nearly natural. Continue morphine.

14th.—Feels very comfortable this morning. Removed the dressings from the wound, which appears to be entirely united. As there was no suppuration, the sutures were not removed. ℞—Continue morphine if necessary.

15th.—Pulse 86 and natural. Passed some blood with her urine, and has some pain in the region of the bladder; otherwise comfortable. ℞—Continue morphine if restless.

16th.—Feels well this morning. Pulse 75; urine normal. Removed the sutures; wound entirely healed, except around the pedicle. Every way comfortable.

17th.—Bowels moved for the first time since the operation, by an enema. Appetite good. She wants to get up, but advised to keep the bed, except to evacuate the bowels or bladder.

23rd.—For the last six days she has continued to improve steadily; has been up a number of times. Pulse 70 or 80; appetite good. Ligatures came away to-day, and the pedicle slipped into the peritoneal cavity, leaving no perceptible opening. Continue adhesive plaster and bandage to support the relaxed abdominal muscles. The bowels being rather constipated, prescribed a dose of castor oil.

24th.—Oil moved the bowels freely. Feels well; appetite good; pulse 75, soft and normal; tongue clean; no disturbance of the bowels.

27th.—I did not visit her for the last three days. She has been steadily gaining strength; appetite good; bowels moved every day, once only; sits up an hour at a time without inconvenience.

30th.—Found her up and dressed, packing her clothes to go home, to Lacon, Marshall Co., Ill. I was surprised, for I warned her not to walk about until I saw her. She said that she felt just as well as ever, and ought to be at home to attend to her affairs. I prevailed on her however to stay until Tuesday, the 5th of August, when she left, feeling every way perfectly well, making 26 days from the time of the operation.

I received a letter from her daughter, dated August 10th, saying that her mother got home well, though was very weak, but that she soon got rested, and was gaining very fast.

Another letter, received August 24th, reports complete re covery.

Remarks.—On examining the reported cases of ovariotomy, we find, that in nearly all of those that prove fatal, that death is caused, either by the *shock* of the operation, hemorrhage either primary or secondary, or inflammation from the injury and exposure of the viscera.

In this case the shock was not at all severe. The patient survived it to admiration. With regard to hemorrhage, not an ounce of blood was lost. Having the pedicle exterior to the wound, we drew the ligatures so tight that the circulation was completely cut off, thereby preventing the possibility of hemorrhage, and hastening the separation by the ligatures. The ecraseur has been recommended to bruise off the pedicle. We had one at hand, but fearing the possibility of hemorrhage, concluded not to use it. The result shows, that in this case at least, it could not have been better than the ligature.

Finally inflammation, which is most to be feared after ovariotomy, was prevented or opposed by keeping the patient on her back, without suffering her to raise up on any condition; evacuating the bladder with the catheter as often as necessary; giving little or no nourishment for the first few days; confining the bowels until the wound was healed; and keeping the patient perfectly quiet and free from every irritating cause.

The two last indications were admirably accomplished with morphine, which, in fact, was the only medicine given, with the exception of a dose of castor oil on the thirteenth day, and a dose of opium immediately after the operation.

